# Using the Neurofibromatosis Tumor Predisposition Syndromes to Understand Normal Nervous System Development

**DOI:** 10.1155/2014/915725

**Published:** 2014-08-28

**Authors:** Cynthia Garcia, David H. Gutmann

**Affiliations:** Department of Neurology, Washington University School of Medicine, Box 8111, 660 South Euclid Avenue, St. Louis, MO 63110, USA

## Abstract

Development is a tightly regulated process that involves stem cell self-renewal, differentiation, cell-to-cell communication, apoptosis, and blood vessel formation. These coordinated processes ensure that tissues maintain a size and architecture that is appropriate for normal tissue function. As such, tumors arise when cells acquire genetic mutations that allow them to escape the normal growth constraints. In this regard, the study of tumor predisposition syndromes affords a unique platform to better understand normal development and the process by which normal cells transform into cancers. Herein, we review the processes governing normal brain development, discuss how brain cancer represents a disruption of these normal processes, and highlight insights into both normal development and cancer made possible by the study of tumor predisposition syndromes.

## 1. Normal Nervous System Development

**Figure 1:**
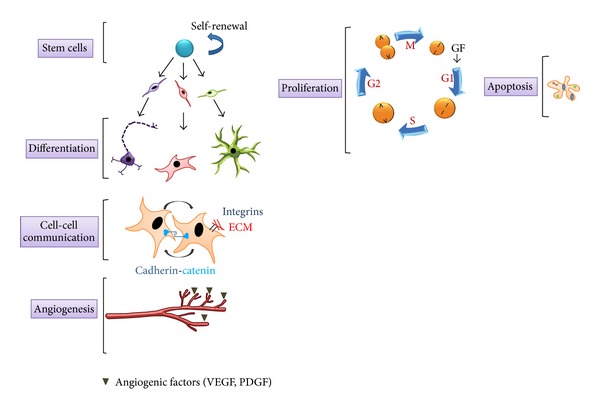
Normal brain development. The nervous system develops from neural stem cells (NSCs) capable of self-renewal, which can divide and generate cells with equal developmental potential. These NSCs also generate differentiated cell types, such as glial-restricted and neuron-restricted progenitors, which further give rise to astrocytes, oligodendrocytes, and neurons. The expansion of progenitors and their progeny is dependent on orderly cell cycle progression and controlled escape from programmed cell death (apoptosis). Developing cells also form cell-cell interactions through cell adhesion molecules, which transmit regulatory signals to influence cell growth and migration. Finally, both progenitor and differentiated cell types actively induce the formation of blood vessels to provide oxygen and nutrients.

The formation of the nervous system requires a highly orchestrated series of events involving cell division, differentiation, death, migration, and intercellular communication (Figure 1). During embryonic and fetal life, the brain and spinal cord originate from a pool of neural stem cells (NSCs) with the capacity to self-renew as well as to give rise to more specialized cells. Self-renewal is the process by which a single stem cell divides to generate one (asymmetric division) or two (symmetric division) daughter stem cells with identical properties as the original mother cell. During cell division, NSCs must enter the cell cycle and replicate their DNA (S phase) prior to forming two daughter cells through mitosis (M phase). During asymmetric division, central nervous system (CNS) NSCs generate restricted progenitor cells (glial or neural progenitors) with more limited differentiative capacity. In this manner, glial progenitor cells give rise to oligodendrocytes or astrocytes, while neural progenitors give rise to neurons. The fate division that dictates the formation of two identical NSCs versus a cell lineage-restricted progenitor is a highly regulated process.

Following cell specialization, there are defined periods of cell migration, in which neurons, astrocytes, and oligodendrocytes (or their progenitors) move to particular positions within the developing brain and spinal cord. The orderly migration of neurons to specific layers (laminae) is essential for the proper organization of the brain and for the formation of functional neuronal connections [1, 2]. Failure to migrate to the right place at the right time can result in the generation of ectopically located neurons, which can cause seizures or cognitive deficits [3, 4].

In addition to cell division and differentiation, there are periods during embryonic development when excess numbers of cells must be culled. This well-ordered process requires the enactment of a programmed cell death pathway (apoptosis). Apoptosis is necessary for the proper spacing and orientation of neurons and for generating spaces like the ones between fingers and toes [5, 6]. During programmed cell death, the nucleus becomes fragmented and the cellular membranes are disrupted, causing the cell to break up into vesicles (apoptotic bodies), which are then removed by other cells by phagocytosis.

Intercellular communication is likewise necessary to establish a functional nervous system, as it provides a way for cells to receive instructive cues from neighboring cells. These instructions can be delivered by soluble molecules (e.g., growth factors and cytokines) or by membrane-bound ligands (e.g., integrins). Soluble cues establish gradients for directed cell migration or signal to recipient cells via plasma membrane receptor tyrosine kinases (RTKs). These RTKs transduce the extracellular signals by activating intracellular signaling pathways through the sequential phosphorylation of downstream effector molecules (e.g., RAF and PI3-Kinase).

Finally, during brain development, progenitor and more differentiated cells actively induce the formation of new blood vessels to provide oxygen and nutrients crucial for cell function and survival. The formation of blood vessels comprises two distinct steps: vasculogenesis, the* de novo* formation of blood vessels, and angiogenesis, the sprouting of preexisting blood vessels. Both vasculogenesis and angiogenesis are elicited by angiogenic factors, such as vascular endothelial growth factor (VEGF) and platelet-derived growth factor (PDGF) that bind to transmembrane RTKs on endothelial cells [7, 8]. During development, there is higher expression of these angiogenic factors, returning to lower levels once mature tissues have formed.

As might be predicted for a highly organized tissue, defects in any of these processes during specific times of brain development can lead to abnormal cell growth (tumors), brain malformations (lissencephaly and heterotopias), or intellectual disabilities (autism) [9–11]. In an effort to identify the key cellular and molecular determinants that underlie normal brain development, researchers have often turned to inherited conditions in which affected individuals exhibit abnormalities in brain development: for example, children with lissencephaly harbor mutations in the* LIS1* gene and are born with a brain malformation characterized by defects in the normal folded architecture of the cortex [12]. Using mouse models of lissencephaly, this brain abnormality (“smooth” cortex) is due to impaired migration of neurons into the developing brain [13, 14]. Similarly, individuals with tuberous sclerosis complex (TSC) develop a wide range of brain pathology, including abnormal collections of neurons (heterotopias), due to a mutation in the* TSC1* or* TSC2* gene [15]. Using mice with brain* Tsc1* gene inactivation, these heterotopias were shown to result from abnormal migration of neural progenitor cells into the rostral migratory stream and olfactory bulb [16, 17] as a direct consequence of increased activation of the Rheb GTPase molecule, a downstream effector of the TSC protein complex [18]. In addition, individuals with a germline mutation in the* PTEN* gene develop Lhermitte-Duclos disease (LDD), a neurological condition characterized by dysplastic gangliocytomas (benign neuronal overgrowths), leading to seizures [19, 20]. Using* Pten* knockout mouse strains, loss of* PTEN* function results in increased granule neuron size, defects in neuronal migration and brain patterning, and abnormalities in synaptic structure due to hyperactivation of the PTEN-regulated protein, protein kinase B (AKT) [21–23]. Finally, Rett syndrome is characterized by microcephaly, autism, and developmental regressions in early childhood as a result of mutations in the* MECP2* gene [24, 25]. Although the exact molecular and cellular mechanisms underlying Rett syndrome pathogenesis have not been completely elucidated, studies employing genetically engineered mice have shown that loss of* MeCP2* function results in decreased dendritic spine density and defects in GABAergic transmission [26, 27].

## 2. Cancer as a Disease of Deregulated Development

**Figure 2:**
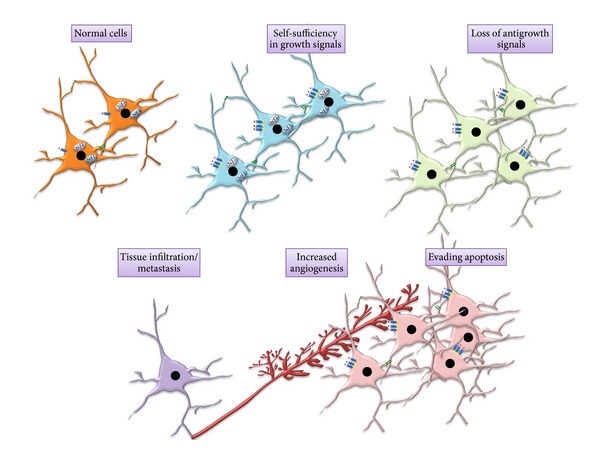
Cancer requires the deregulation of several normal developmental processes. Cells acquire at least five alterations that collectively lead to cancer: self-sufficiency in growth signals, loss of antigrowth signals, evasion of apoptosis, increased angiogenesis, and inappropriate tissue invasion. Tumor development begins when a cell (either a stem cell or a differentiated cell) acquires a mutation that increases its propensity to proliferate and decreases its ability to undergo apoptosis. Cell-cell interactions become subsequently deregulated to allow cancer cells to promote angiogenesis and tissue invasion (metastasis).

There are many reasons to view cancer as a disease of abnormal development. Cancer requires that the cellular processes essential for normal nervous system development become deregulated (Figure 2). In many tissue types in the body, secreted signaling (paracrine) factors have been identified that either stimulate or inhibit progression through the cell cycle. Mitogenic paracrine factors (growth factors) act at the G_1_ to S-phase transition of the cell cycle by binding to their cognate growth factor receptors. Growth factor receptor engagement leads to conformational changes in the receptors and phosphorylation of specific residues in the intracellular portions of the molecule to promote the binding and activation of downstream signaling intermediates [28, 29]. Whereas normal cells divide in response to growth factor stimulation, tumor cells liberate themselves of these constraints in one of four ways. (1) Cancer cells may acquire the ability to synthesize their own growth factors, such as epidermal growth factor (EGF) and PDGF [30, 31], to stimulate growth in an autocrine fashion. (2) Neoplastic cell types may overexpress a particular growth factor receptor [32, 33] or express one with a constitutively active mutation [34, 35], leading to enhanced responsiveness to available growth factors or growth factor-independent growth, respectively. (3) Cancer cells can also acquire self-sufficiency through abnormal expression or function of integrins [36]. Integrins are transmembrane receptors that link the extracellular matrix (ECM) to the cytoskeleton, transmitting both mechanical and chemical signals. Whereas normal cells employ integrins to remain in a quiescent state [37, 38], cancer cells with dysfunctional integrin signaling allow for enhanced survival, proliferation, and migration [39–41]. (4) The effector proteins downstream of RTKs or integrins can be mutationally activated to lead to constitutive growth signaling in the absence of growth factors or integrins. Examples of these protooncogenes, including Ras, Src, and Raf, have all been mutated in human cancers [42–44]. Each of these protooncogenes, when mutationally activated, can initiate cancer in experimental model systems [45–47].

Another reason to view cancer as a disease of development is that tumor cells sometimes disable the proteins that negatively control normal cell growth. This escape can be mediated in part through loss of tumor suppressor gene function. Tumor suppressors inhibit cell proliferation by suppressing protooncogene function, thus preventing them from activating their downstream effector proteins to promote cell growth. In this manner, PTEN negatively controls AKT activity, such that loss of* Pten* gene expression results in increased AKT signaling and cell growth. In addition, tumor suppressor proteins control the function of cyclins required for orderly cell cycle progression, and their mutational inactivation (e.g., retinoblastoma) deregulates cyclin function and leads to higher levels of proliferation [48, 49].

Moreover, tumor growth may also result from an escape from normal programmed cell death. Activation of either of the two apoptotic pathways (extrinsic and intrinsic) can initiate cell death through the sequential activation of caspases (cysteine-aspartic acid proteases). Whereas the engagement of the extrinsic pathway requires ligand activation of death receptors, the intrinsic pathway involves Bcl-2 family member-mediated mitochondrial cytochrome-c release [50]. Engagement of the apoptotic machinery is observed following mutational inactivation of the* TP53* tumor suppressor gene; however, mutations in negative regulators of the PI3-Kinase/AKT pathway also provide prosurvival signals by inhibiting Bcl-2 family member function [51]. As such, loss of function mutations in the* PTEN* and* NF1* tumor suppressor genes leads to increased AKT activity and attenuated apoptosis [52, 53].

During embryogenesis, cells obtain oxygen and nutrients from the developing vasculature, formed in response to angiogenic factors like VEGF and PDGF [54, 55]. Similar to development, tumors are maintained by the formation of new blood vessels (neoangiogenesis). In this regard, cancer cells elaborate the same angiogenic factors [56] that produce blood vessel growth in the embryo to promote increased oxygen and nutrient delivery.

Another acquired property of tumors is tissue invasion, through both local spread (infiltration) and distant seeding (metastasis). In order to accomplish this, neoplastic cells must undergo a cascade of events that allow the cells to leave the primary tumor site. First, cells develop alterations in their shape as well as attachment to one another and to the ECM through loss of cell-to-cell interactions and/or cell-to-ECM adhesion molecules. Loss of cell-to-cell adhesion allows dissociation from the original tumor, enhanced cell locomotion, and infiltration of surrounding tissue. Disrupted cell-to-cell adhesion and infiltration have been shown in E-cadherin and N-cadherin knockout mouse models [57, 58]. Similarly, adhesion molecules associated with cell migration during embryogenesis also facilitate cell migration and infiltration in neoplastic cells when deregulated. For example, overexpression of N-cadherin in breast cancer cells promotes increased cell motility and infiltration [59]. In addition, integrins also associate with RTKs to activate signaling pathways necessary for cell migration. For metastasis to occur, tumor cells must enter blood vessels, at which point they travel to distant sites, and then exit the blood vessels to seed these new sites. This process involves paracrine signaling between RTKs (e.g., colony stimulating factor-1 receptor: CSF1R) on macrophages and EGFR on tumor cells [60]. A final step in cancer malignancy involves the outgrowth of these secondary tumors at these remote sites. There are at least twelve known metastasis suppressor genes that inhibit invasion and metastasis, including the Raf kinase inhibitor protein (RKIP) molecule, the dysfunction of which promotes invasion and metastasis of cancer cells in a Raf/MEK-dependent manner [61, 62].

Beyond these cell intrinsic factors, other elements also contribute to tumor formation, including biophysical forces [63], endocrine disruptors [64], and non-neoplastic cells in the tumor microenvironment [65, 66]. The importance of these stromal cells (macrophages, endothelial cells, and reactive astrocytes) to nervous system tumorigenesis as well as tumor maintenance has led to the identification of numerous cytokines and growth factors [67, 68] critical to the genesis and growth of these cancers. As such, the non-neoplastic cell types and growth signals that exist in the tumor microenvironment represent logical targets for therapeutic drug design [69, 70].

In the following sections, we will use two nervous system tumor predispositions, neurofibromatosis type 1 (NF1) and neurofibromatosis type 2 (NF2), to illustrate the overlap between cancer and normal development and to demonstrate how these inherited syndromes provide instructive insights for developmental neurobiologists and neuro-oncologists alike.

## 3. Neurofibromatosis Type 1

NF1 is an autosomal dominant inherited cancer predisposition syndrome that affects 1 in 2,500 individuals worldwide [71]. Similar to other inherited cancer syndromes [72], individuals with NF1 are born with a mutated (nonfunctional) copy of the* NF1* gene in all cells in their body. Tumors arise following somatic gene inactivation of the remaining functional* NF1* allele in specific cell types, leading to complete loss of neurofibromin function in those cells. As would be expected from a cancer predisposition syndrome, individuals with NF1 are prone to the development of benign and malignant tumors, in this case, affecting the peripheral nervous system (PNS) and central nervous system (CNS). As such, people with NF1 develop optic pathway gliomas, neurofibromas, and malignant peripheral nerve sheath tumors [73–75]. Optic pathway gliomas are glial cell tumors detected in about 15% of children with NF1, typically in individuals younger than seven years of age [73]. When symptomatic, optic pathway gliomas cause impairments in vision or blindness.

Neurofibromas are benign Schwann cell tumors that begin to appear during adolescence, arising anywhere in the body as cutaneous, subcutaneous, or deeply located masses [76]. A more diffuse type of neurofibroma, the plexiform neurofibroma, is often detected in very young children, where they can grow to significant proportions and cause morbidity through compression of surrounding tissues [77]. Less commonly, plexiform neurofibromas transform into malignant peripheral nerve sheath tumors (MPNSTs) [78]. These sarcoma-type cancers are highly metastatic and typically unresponsive to conventional therapies [79].

## 4. *NF1* Gene Structure and Function

**Figure 3:**
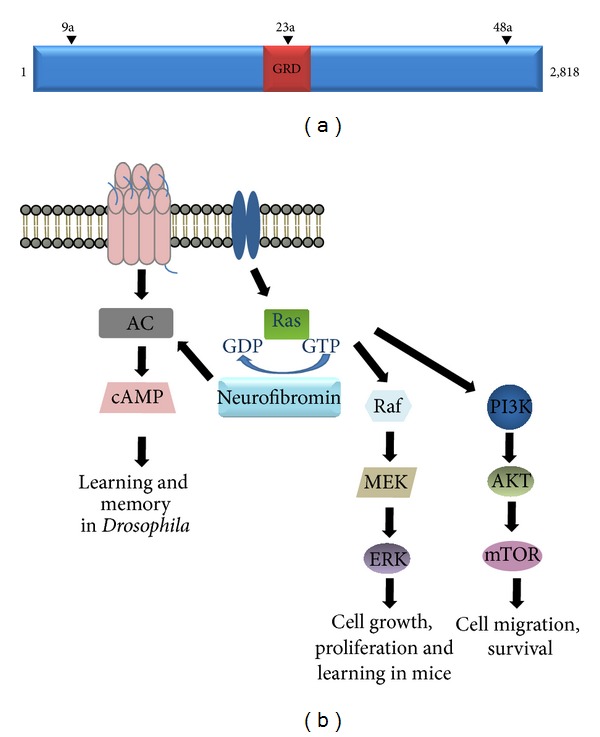
Neurofibromin structure and function. (a) Neurofibromin is a 2,818-amino-acid cytoplasmic protein with three alternatively spliced exons (9a, 23a, and 48a) and a central GAP-related domain (GRD). GAPs are negative regulators of RAS by accelerating the conversion of active GTP-bound RAS to its inactive GDP-bound form. (b) Loss of neurofibromin function leads to increased RAS pathway (RAF/MEK/ERK or PI3K/AKT/mTOR) signaling as well as reduced cAMP generation.

The* NF1* gene is located on chromosome 17q11.2 and codes for a 2,818-amino-acid protein called neurofibromin [80] (Figure 3(a)). Neurofibromin is a large cytoplasmic molecule with three differentially spliced exons (9a, 23a, and 48a) that may be important for neurofibromin function in specific cell types [81–85]. Inspection of the predicted coding sequence reveals that neurofibromin contains a small 300-amino-acid domain with significant homology to guanosine triphosphatase- (GTPase-) activating proteins (GAPs) involved in the negative regulation of the RAS small GTPase proto-oncoprotein. In this regard, neurofibromin negatively regulates RAS activity by accelerating the hydrolysis of active GTP-bound RAS to inactive GDP-bound RAS [86].

In NF1-associated tumors, neurofibromin loss leads to Ras hyperactivation and increased signaling through the Ras downstream effector proteins, RAF/MEK (ERK pathway) and PI3-Kinase (PI3K)/AKT (Figure 3(b)). While ERK has been implicated in cell proliferation control, AKT signaling is important for promoting cell migration and survival. Both pathways have been implicated in* Nf1*-deficient cell growth and proliferation [53, 87–89]. Furthermore, neurofibromin is also an important regulator of cyclic AMP (cAMP), which has been implicated in learning and memory. Studies in* Drosophila* have shown that the* Nf1* gene is required for activation of the adenylyl cyclase/cAMP pathway, which mediates learning and memory [90]. In mice, decreased cAMP activity due to neurofibromin loss or reduction results in increased astrocyte proliferation [91] as well as attenuated CNS axon length and survival [92].

Hyperactivation of the signaling pathways that govern NF1-associated tumor growth can result from growth factors or cytokines produced by non-neoplastic cells in the tumor microenvironment. The dependence on these stromal cells and growth factors is nicely illustrated by* Nf1* mouse models of plexiform neurofibroma and optic glioma [93]. In these genetically engineered mouse strains, loss of* Nf1* gene expression in neoplastic Schwann cell or neuroglial progenitors alone is not sufficient for plexiform neurofibroma [94] or optic glioma [95, 96] formation, respectively. Instead, tumorigenesis requires complete* Nf1* inactivation in progenitors to be coupled with reduced* Nf1* gene expression (*Nf1*+/− mice). Whereas plexiform neurofibromas require* Nf1*+/− mast cells [97, 98], optic glioma formation and continued growth are dependent on* Nf1*+/− microglia [99–101]. The growth factors produced by these stromal cells have recently been targeted in preclinical mouse studies with encouraging early results [98, 102].

## 5. Neurofibromin Function during Normal Nervous System Development

**Figure 4:**
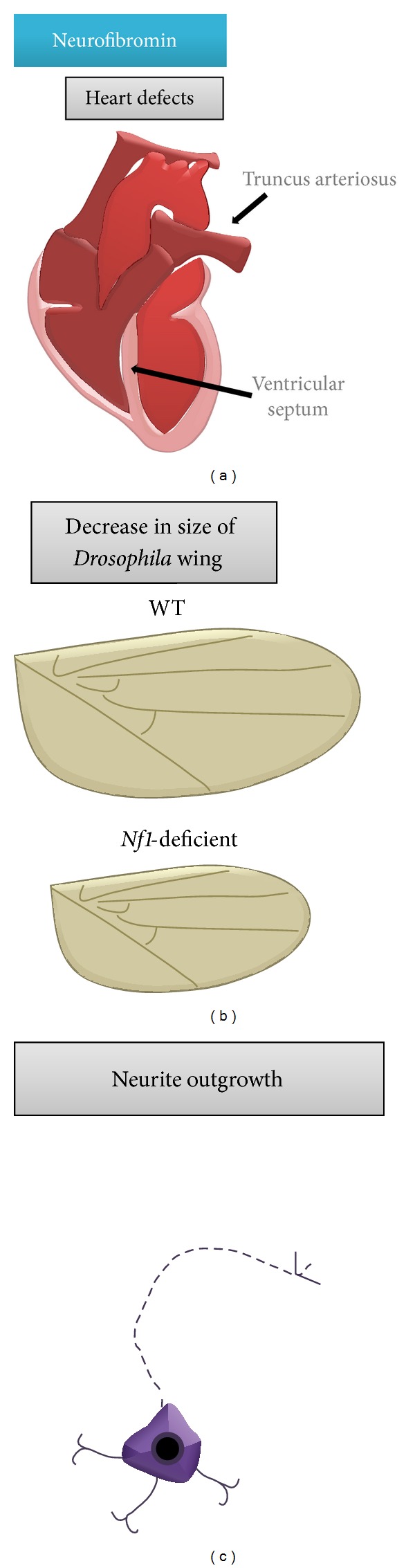
Neurofibromin function during normal development. Neurofibromin is important for proper (a) heart vessel development, (b)* Drosophila* wing formation (cell proliferation and size), and (c) axon extension and neuron survival.

The critical role of the* Nf1* gene in normal development is underscored by several observations made in genetically engineered mice. Complete loss of neurofibromin in mice results in embryonic lethality between days E12.5 and E13.5. The cause of death in these* Nf1*-deficient embryos is the consequence of a defect in cardiac vessel formation (Figure 4(a)). Instead of having a separate aorta and pulmonary artery, these mice have a single outflow tract, known as double outlet right ventricle [103]. This defect is caused by abnormal* Nf1*-deficient cardiac neural crest cell migration.

Owing to the lethality that results from complete* Nf1* gene inactivation in mice, insights into the importance of neurofibromin during development have derived from the use of* Nf1* mutant flies and* Nf1* conditional knockout mice (CKO). Loss of* Nf1* gene expression in* Drosophila* results in a 25% reduction in wing size (Figure 4(b)) and in the overall size of larvae, pupae, and adults [104]. This small size defect is not restored by manipulating RAS signaling but rather is rescued by overexpression of activated protein kinase A (PKA). The rescue by PKA, and not by RAS, supports the notion that neurofibromin positively regulates cAMP independent of RAS and that some of the NF1-associated phenotypes may reflect reduced cAMP levels. Studies in mice have also shown that neurofibromin positively regulates cAMP activity [91, 105]. In this regard, the use of* Nf1* CKO mice in which neurofibromin loss occurs in BLBP^+^ progenitor cells or* Nf1*+/− primary CNS neurons has demonstrated that neurofibromin is critical for axonal extension and neuronal survival in a cAMP-dependent manner (Figure 4(c)) [92, 106, 107].

In addition, neurofibromin is an important regulator of NSC function [107, 108]. As such,* Nf1* gene inactivation results in increased proliferation of spinal cord progenitors [109], brainstem NSCs [89], and telencephalic NSCs [108]. The mechanism underlying neurofibromin regulation of CNS NSC function involves control of RAS signaling.

Lastly, neurofibromin is also important for neuronal and glial differentiation in the brain. In this regard,* Nf1* CKO mice in which the* Nf1* gene is inactivated in E12.5 GFAP^+^ cortical progenitors exhibit a significant reduction in patterning of cortical cells into whisker barrels throughout the somatosensory cortex [110]. This patterning defect was shown to result from increased RAS/ERK signaling. Similarly,* Nf1* CKO mice in which* Nf1* is inactivated in neurons have reduced cortical thickness [111]. In contrast, neurofibromin regulates brain glial differentiation (gliogenesis) in a RAS/mammalian target of rapamycin (mTOR)-dependent manner [89, 107].

## 6. Neurofibromatosis Type 2

NF2 is also an autosomal dominant inherited cancer predisposition syndrome, caused by a germline mutation in the* NF2* gene. Whereas the hallmark of this disorder is bilateral vestibular (VIIIth cranial nerve) schwannoma development, individuals with NF2 also harbor other cranial and peripheral nerve schwannomas, meningiomas, and spinal ependymomas. Teenagers and adults may come to medical attention when they present with hearing loss, balance problems, weakness, or seizures secondary to tumor development and progression. Similar to other cancer predisposition syndromes, tumors arise following somatic* NF2* gene inactivation in specific cell types to render both copies of the* NF2* gene nonfunctional in those cells [112, 113]. The importance of the* NF2* gene to nervous system tumor formation is further underscored by the finding that* NF2* locus alterations predominate in sporadic (non-NF2-related) schwannomas, meningiomas, and ependymomas [114–118]. Collectively, these clinical observations indicate that the* NF2* gene is a key regulator of normal cell function in Schwann cells, meningeal cells, and spinal ependymal cells.

## 7. *NF2* Gene Structure and Function

**Figure 5:**
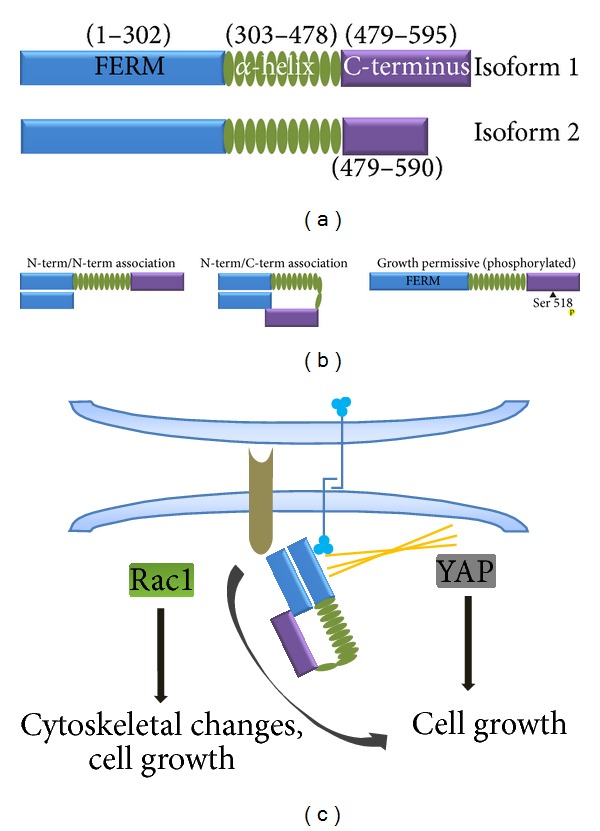
Merlin structure and function. (a) Merlin is a 595- (or 590-) amino-acid protein composed of three structural regions: an amino terminal domain (FERM), an α-helix domain, and a carboxyl terminal domain (CTD). There are two merlin isoforms. Isoform 1 contains exon 17 and lacks exon 16. Isoform 2 contains exon 16, which inserts 11 unique c-terminal amino acids followed by a termination codon that prevents the translation of exon 17. (b) Merlin forms an intramolecular complex in which the FERM domain associates with the CTD. Phosphorylation of merlin at Serine-518 results in an open conformation, which inactivates the tumor suppressor activity of merlin. (c) Merlin binds to the actin cytoskeleton, RTKs, and CD44 to form proper adherens junctions. It also inhibits RAC1 and YAP activity, which are important for normal growth regulation.

The* NF2* gene is located on chromosome 22q and encodes a 595-amino-acid tumor suppressor protein called either merlin or schwannomin [119, 120] (Figure 5(a)). The predicted merlin protein sequence reveals three structural domains, including an amino terminal FERM (four-point one, ezrin, radixin, and moesin) domain (residues 1–302), a central alpha helical region (residues 303–479), and a carboxyl terminus region (480–595). Based on this predicted structure, merlin belongs to the band 4.1 superfamily of proteins that link the actin cytoskeleton to cell surface glycoproteins [121]. There are two isoforms of merlin that differ in their alternative use of exon 16 (isoform 2; missing exon 17) versus exon 17 (isoform 1; missing exon 16) [122, 123]. Whereas merlin isoform 1 is critical for growth regulation and tumorigenesis [124], recent studies have shown that merlin isoform 2 has a key role in peripheral nerve function [125, 126].

Merlin has been hypothesized to exist in either a closed, active state or an open, inactive state mediated by FERM domain/carboxyl terminal domain (CTD) intramolecular binding (Figure 5(b)). Phosphorylation of merlin at several residues, including Serine-518, is thought to inhibit FERM/CTD binding, resulting in the open, inactive configuration. As such, merlin functions as a tumor suppressor in the closed, active state, when the CTD can associate with the FERM domain [124, 127, 128]. While this model may explain merlin growth regulation, other mechanisms must be operative in mediating merlin isoform 2 function.

Unlike neurofibromin, the precise mechanism by which merlin regulates cell growth and motility has not been fully solved. The divergent findings made in different laboratories using different model systems may reflect tissue- or cell type-specific roles for merlin (Figure 5(c)). Merlin functions as a regulator of cytoskeletal dynamics, whereas in other cell types, it regulates the formation of adherens junctions [129] and controls cell motility [130]. For example, complete* Nf2* gene inactivation in mouse embryo fibroblasts (MEFs) results in defective cadherin-mediated intercellular (cell-to-cell) adhesion, such that *β*-catenin, a component of adherens junctions, is no longer localized along cell-cell borders [129]. This merlin-regulated contact-mediated growth arrest occurs by coupling cell surface adhesion molecules (e.g., E-cadherin) to transmembrane RTKs (e.g., the EGF receptor). In this manner, merlin strengthens cadherin-mediated intercellular attachments to control growth factor receptor internalization and mitogenic signaling [131, 132]. The ability of merlin to suppress growth factor receptor function also operates at the level of receptor activation (e.g., ErbB2) [133] or by regulating the abundance or availability of growth factor receptors at the cell surface [134]. While the precise mechanism underlying merlin regulation of cytoskeletal dynamics or adherens junction signaling has not been completely elucidated, merlin negatively regulates the activity of Rac1 [128, 135], a small GTPase protein important for actin assembly and lamellipodia formation [136].

Moreover, merlin also functions as a negative regulator of Hippo/Yes-associated protein (YAP) signaling. When active, the transcriptional activator YAP interacts with the adherens junction protein, α-catenin [137]. However, following pathway activation, YAP translocates to the nucleus to induce the expression of proteins involved in cell cycle progression [138, 139]. Because merlin regulates the formation of adherens junctions, it is possible that loss of merlin function results in destabilized adherens junction structures and leads to the nuclear translocation of YAP and the promotion of cell growth. Consistent with this model, merlin negatively regulates cell growth by inhibiting YAP activation both* in vitro* [140] and* in vivo* [141].

## 8. Merlin Function during Normal Nervous System Development

**Figure 6:**
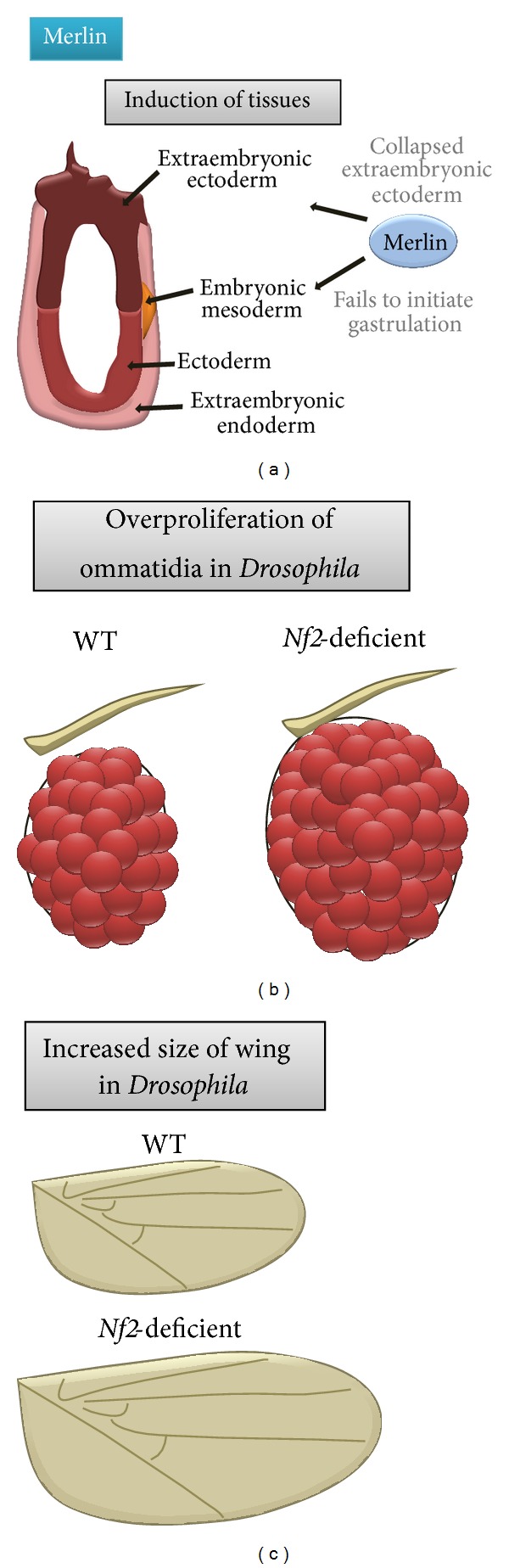
Merlin function during normal development. (a) Merlin is required for the induction of tissue layers during early embryonic development. In addition, merlin is essential for normal control of ommatidia proliferation (b) and (c) wing size in* Drosophila*.

The importance of merlin to normal development has been revealed by studies demonstrating that mouse embryos homozygous for an inactivating* Nf2* gene mutation die during early embryonic development (E6.5–7.0) [142]. These mice exhibit a collapsed extraembryonic region and a disorganized extraembryonic ectoderm [142] (Figure 6(a)). In addition,* Nf2* knockout mice lack expression of mesodermal markers, supporting a defect in the initiation of gastrulation [142].

In* Drosophila*, merlin is a critical regulator of the structural elements of the developing eye (ommatidia), such that loss of merlin results in increased ommatidia cell proliferation [143] (Figure 6(b)). Moreover, these* Nf2* mutant flies have increased overall wing size (Figure 6(c)). The observation that* Nf2*-deficient cells exhibit increased BrdU incorporation and a change in phosphohistone H3 and cyclin E expression [144] supports a defect in growth regulation during development.

Similar to neurofibromin, merlin is also important for normal NSC function. As such, merlin is essential for maintaining the hematopoietic stem cell (HSC) niche. Inactivation of the* Nf2* gene in HSCs results in increased proliferation, owing to increased bone marrow vascularity [145]. Merlin also functions as a negative regulator of liver progenitor cell function. Liver-specific* Nf2* gene deletion leads to increased liver progenitor cell proliferation [146]. In the CNS, merlin inhibits the proliferation of neural progenitor cells (NPCs) in the dorsal telencephalon. Using a CKO mouse in which the* Nf2* gene was deleted in telencephalic EMX1^+^ cells, merlin loss leads to NPC overproliferation, causing a thicker neocortex [147]. In these mice, neuronal differentiation was reduced, leading to a significant reduction in the size of the hippocampus in a YAP-dependent manner. These findings support the notion that proper merlin function is necessary to limit the expansion of NPCs and promote the differentiation of hippocampal progenitor cells.

In the peripheral nervous system, merlin has been shown to be a negative regulator of Schwann cell myelination. Using* Nf2* Schwann cell CKO mice, increased Rac1 activation in Schwann cells, a signaling pathway previously implicated in normal myelination, was observed [148–151]. This defect caused smaller sciatic nerves, resulting in hindlimb dysfunction [151]. In addition, merlin isoform 2 has a unique function in maintaining peripheral axonal integrity by modulating Ras homolog gene family member A (RHOA) activity [126], further demonstrating both isoform and tissue-specific requirements for merlin during normal development.

## 9. Conclusions

Normal brain development involves a rigorously coordinated series of cellular processes, including properly regulated cell growth, balanced by cell proliferation and cell death, coupled with cell differentiation, migration, and connectivity, which are all essential for the initial formation and life-long maintenance of the mammalian nervous system. Disruptions in any of these tightly controlled events could have devastating consequences. In this manner, inappropriate stem cell (progenitor) expansion, glial lineage differentiation, and tissue invasion could set the stage for tumor initiation and malignant progression. By identifying the extracellular cues and intracellular signal transduction pathways that govern normal nervous system development, there is a unique opportunity not only to understand the healthy brain, but also to define how their deregulation contributes to tumorigenesis. The bidirectional information flow between insights derived from developmental neurobiology and those from neuro-oncology creates cross-informative platforms for increasing basic science knowledge and facilitating clinical translation. Leveraging the differences between brain cancer and normal brain homeostasis will be important in designing future effective treatments that target the tumor but spare the normal central nervous system.
